# Supplementary figures and images for: Consistency of VDJ Rearrangement and Substitution Parameters Enables Accurate B Cell Receptor Sequence Annotation
Source: PLoS Comput Biol. 2016 Jan 11;12(1):e1004409. doi: 10.1371/journal.pcbi.1004409 (PMC4709141; doi:10.1371/journal.pcbi.1004409)

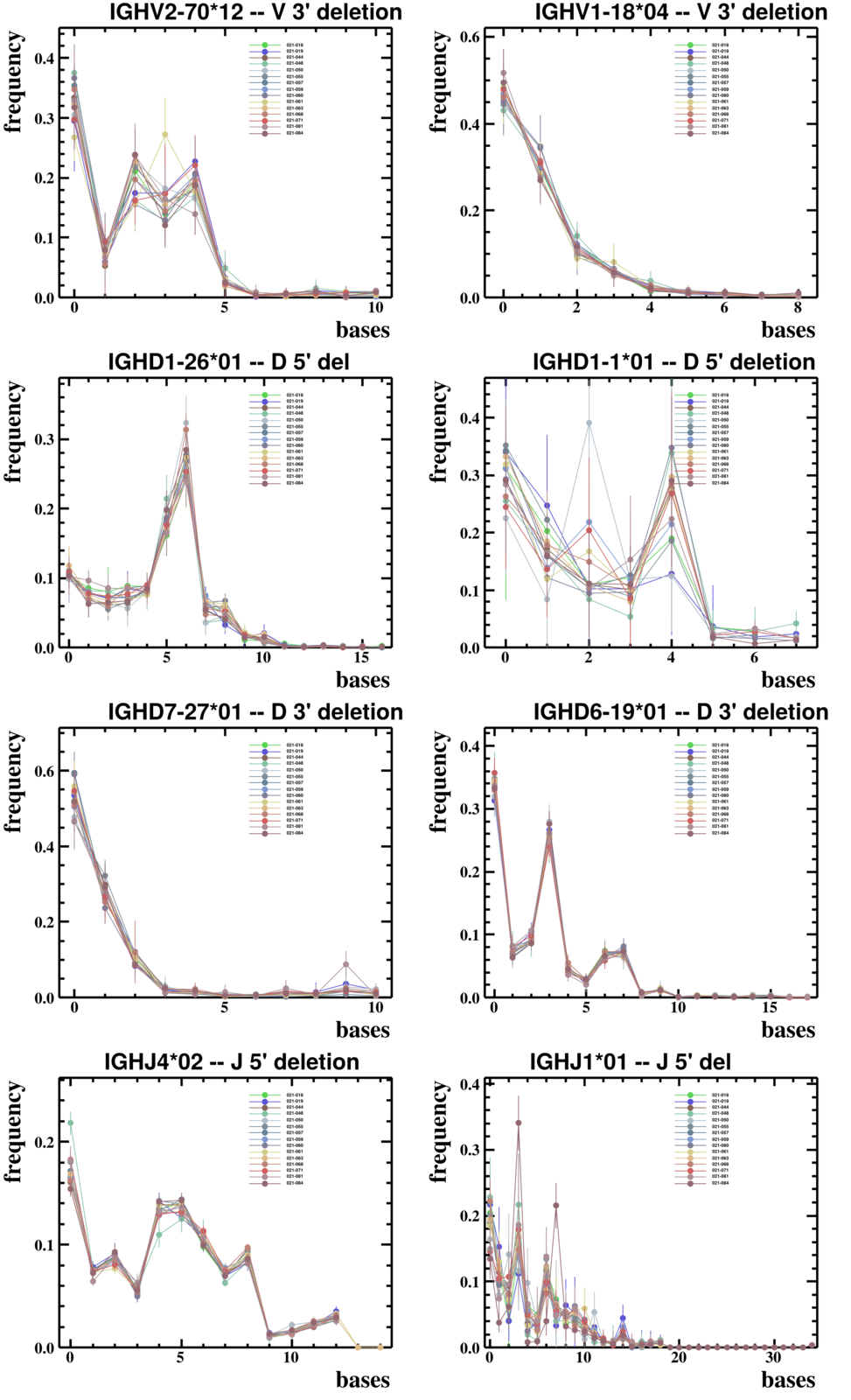

Supplement: S1 Fig — Typical observed exonuclease deletion length frequencies for two V, four D, and two J alleles on the Vollmers data set. (TIFF) [file pcbi.1004409.s001.tiff]

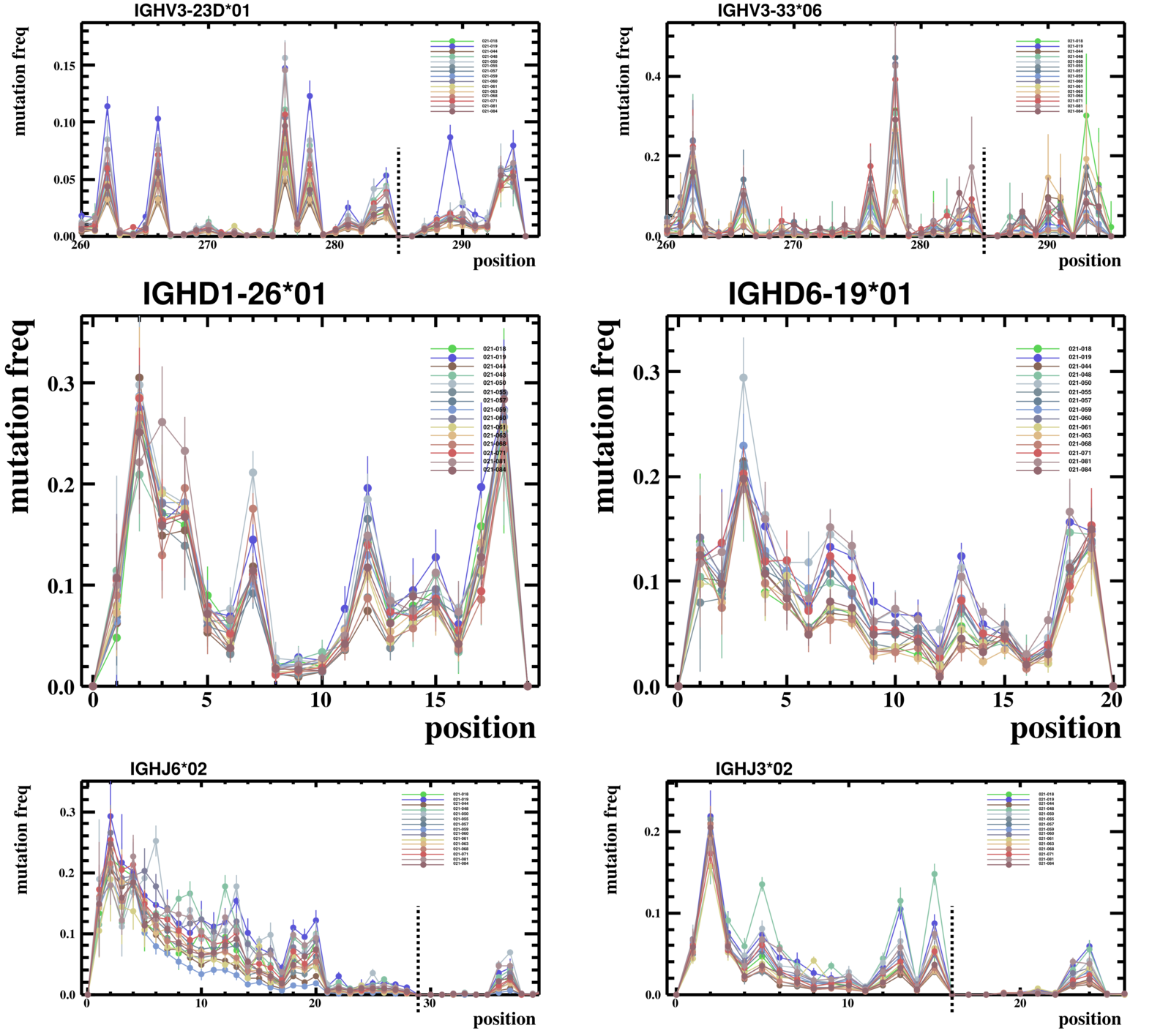

Supplement: S2 Fig — Typical observed mutation frequencies for two V, two D, and two J alleles on the Vollmers data set. The first base of the conserved cysteine and tryptophan codons (i.e. the CDR3 boundaries) are indicated with black vertical dashed lines. The large uncertainties at the 5’ end of V and 3’ end of J reflect that our reads very rarely extend into these regions. Here and elsewhere we use the standard naming convention for alleles of germline genes: for example “IGHV3-33*06” means the 6th allele of the 33rd gene in the V3 gene family for the heavy chain. (TIFF) [file pcbi.1004409.s002.tiff]

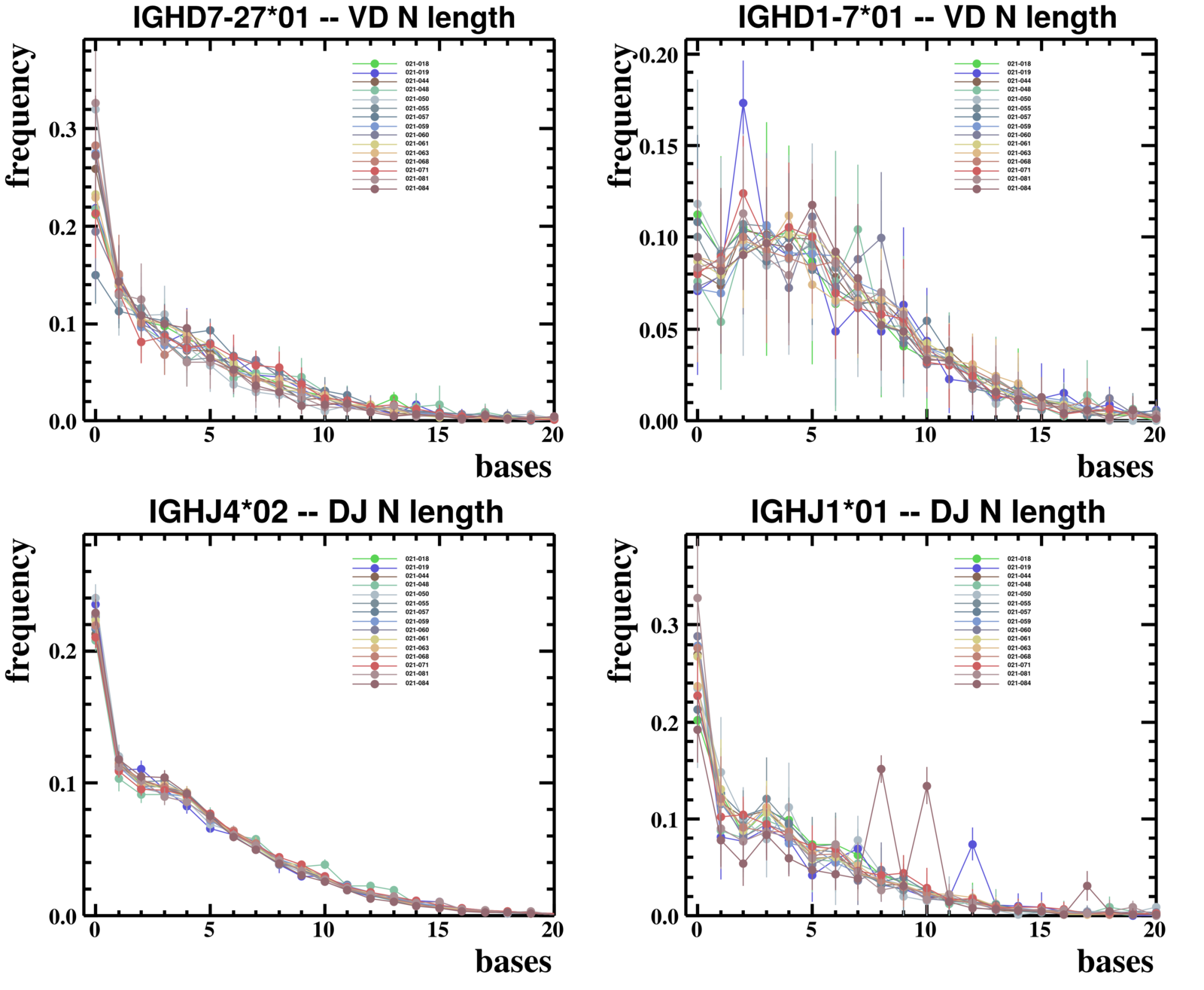

Supplement: S3 Fig — Typical observed N-region lengths at the VD and DJ boundaries for two D and two J alleles. (TIFF) [file pcbi.1004409.s003.tiff]

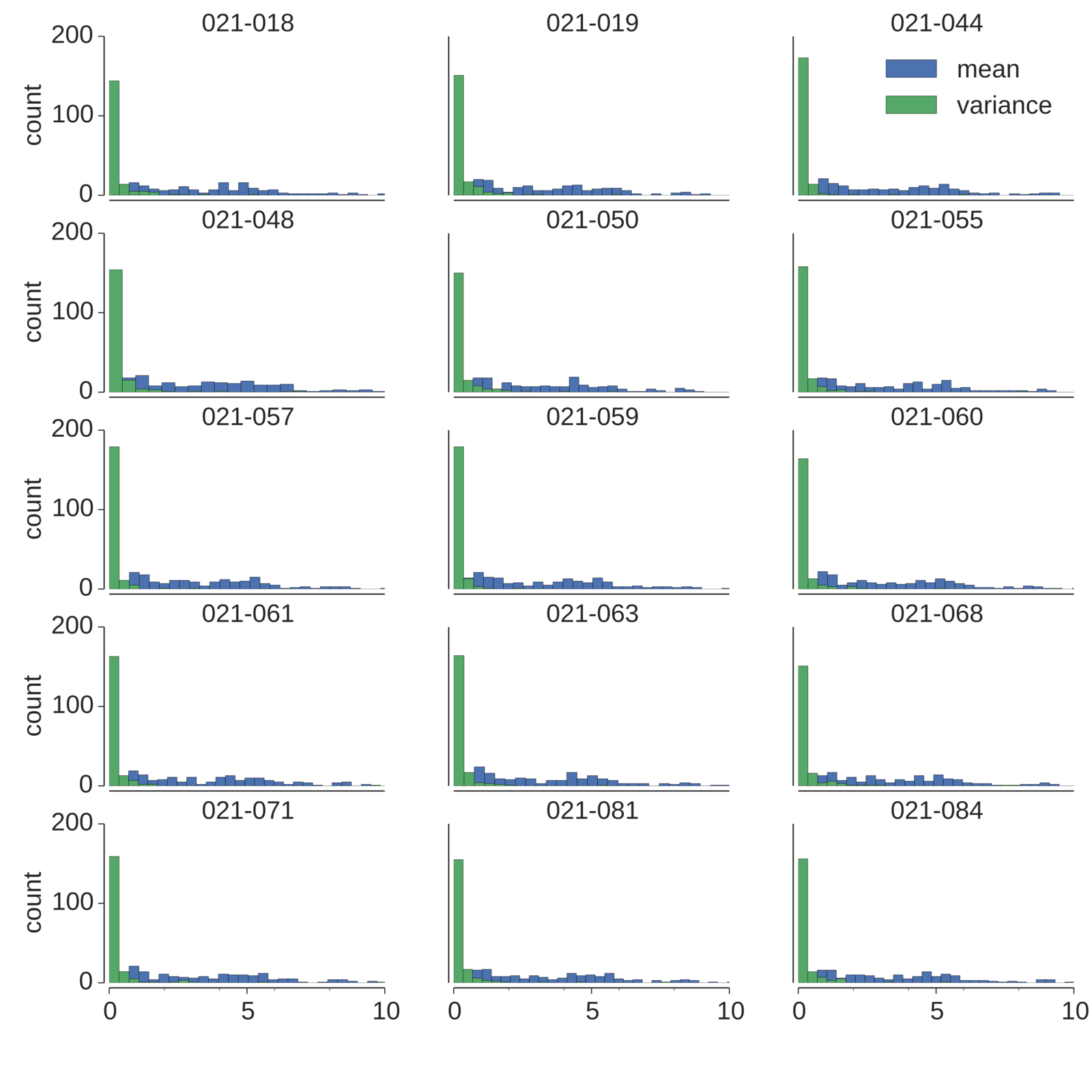

Supplement: S4 Fig — The across-subset mean and variance of inferred parameter values for each human in the Vollmers data set across 10 disjoint subsets of the data. See the caption to Fig 5 and the corresponding text for more details. (TIFF) [file pcbi.1004409.s004.tiff]

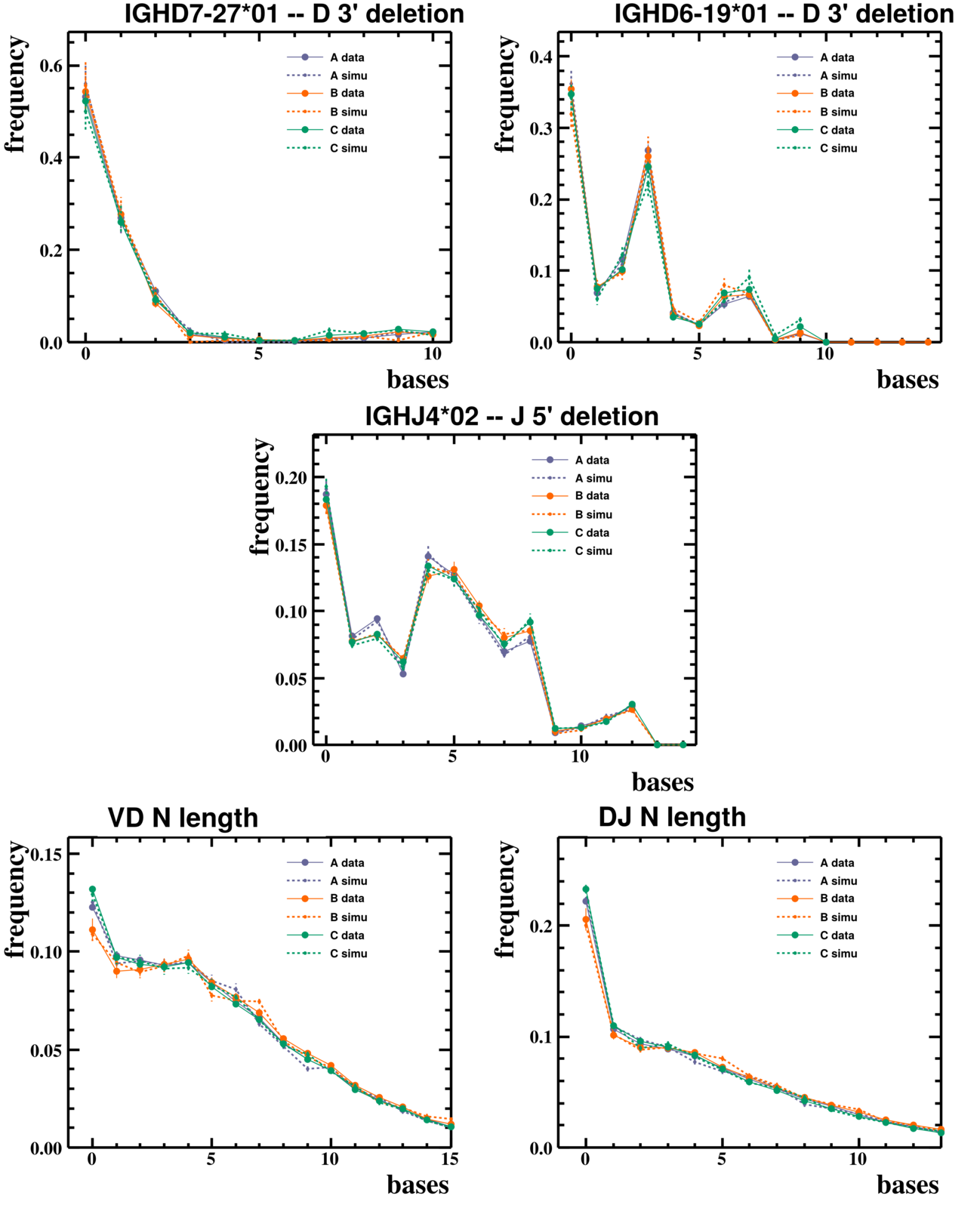

Supplement: S5 Fig — (TIFF) [file pcbi.1004409.s005.tiff]

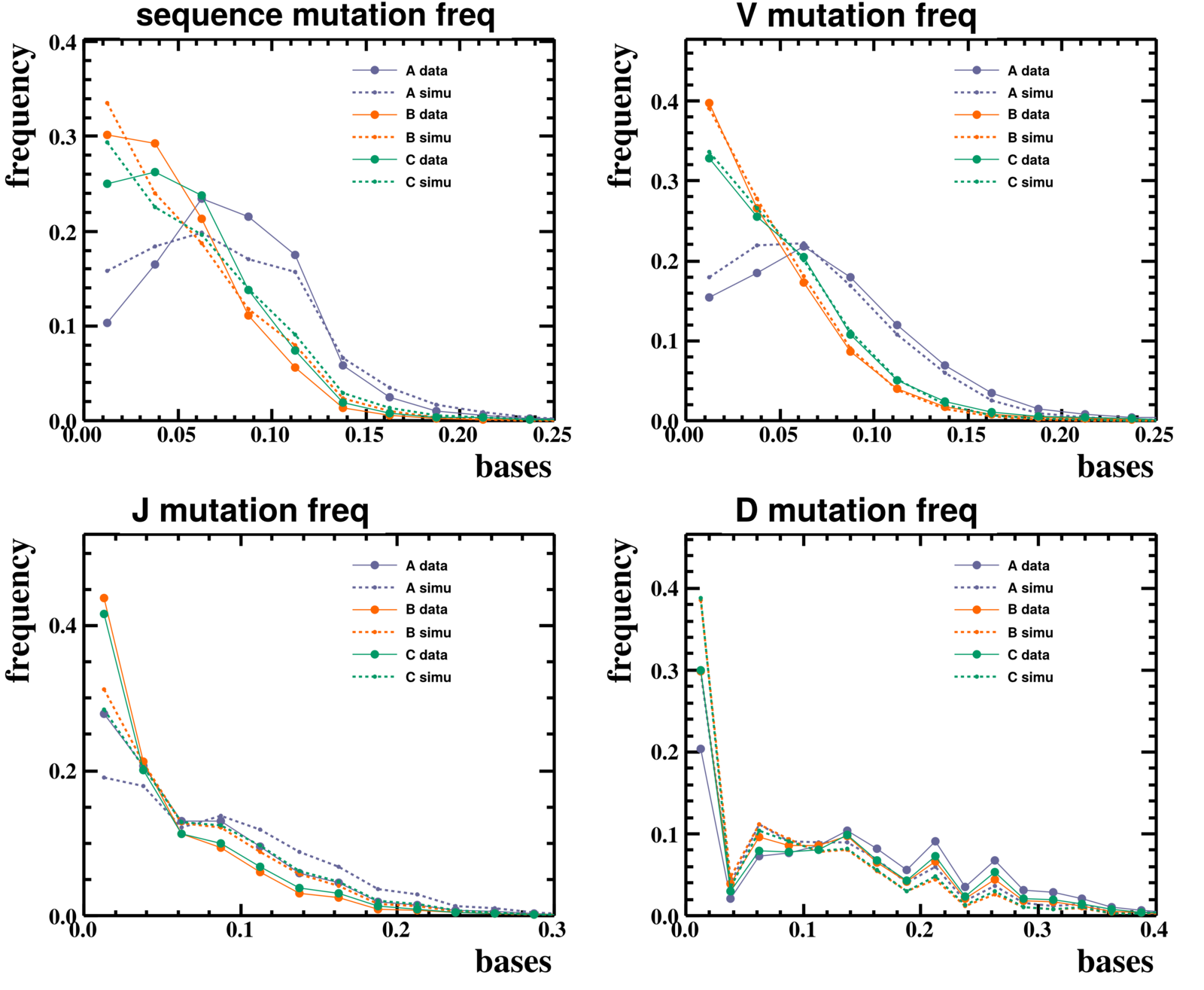

Supplement: S6 Fig — (TIFF) [file pcbi.1004409.s006.tiff]

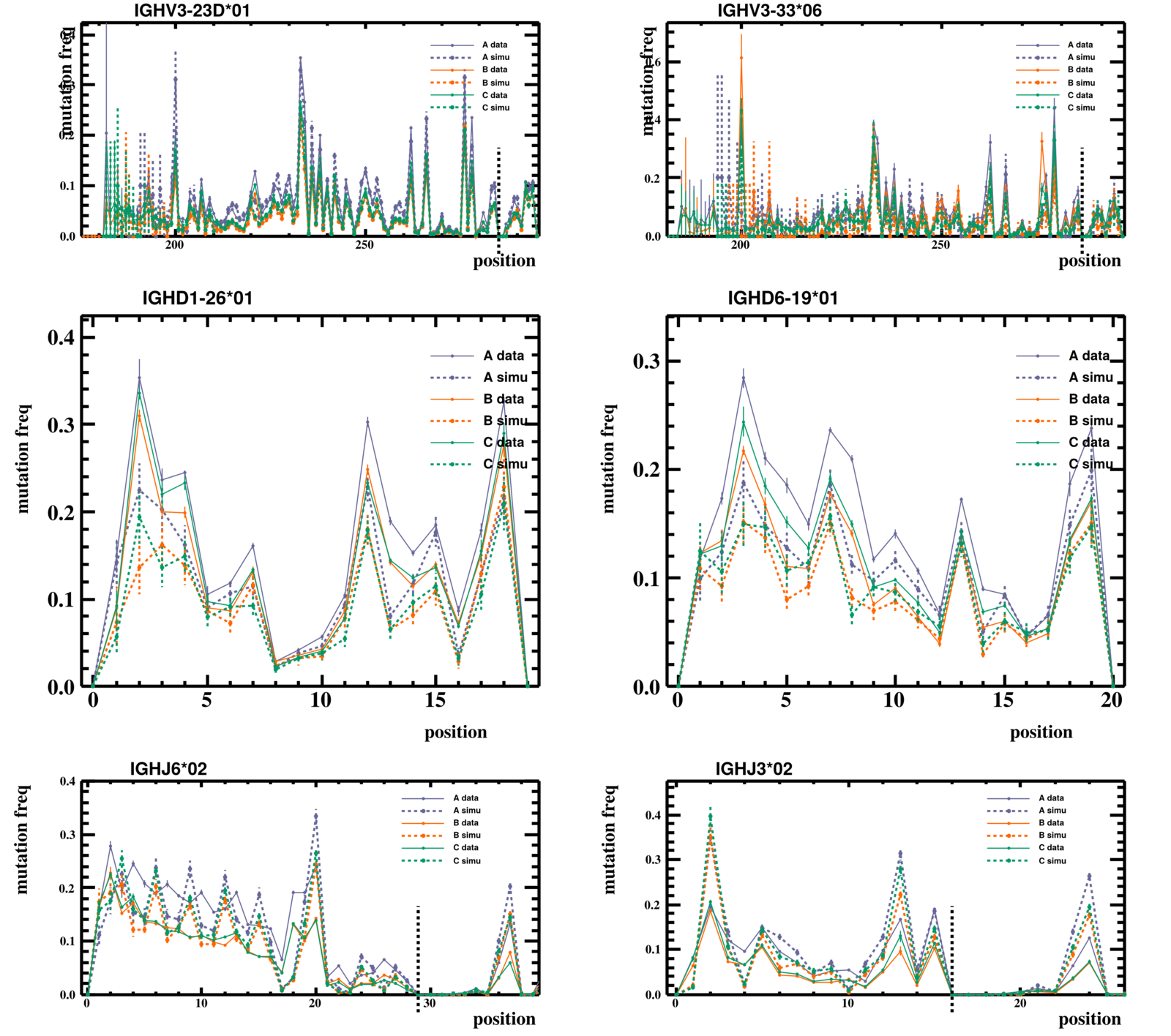

Supplement: S7 Fig — (TIFF) [file pcbi.1004409.s007.tiff]
